# Protective Effects of Exogenous Donkey Oil on Skin Healing Under Incisional Wound Damage

**DOI:** 10.1111/jocd.70550

**Published:** 2025-11-18

**Authors:** Jie Yu, Lili Hu, Jie Cheng, Guangyuan Liu, Min Li, Wei Zhao, Jianping Huang, Hang Tie, Tao Wu, Hongyang Zhang, Bingguang Huang

**Affiliations:** ^1^ Dong‐E E‐Jiao Co. Ltd Liaocheng Shandong China; ^2^ Tianjin Fourth Central Hospital Tianjin China; ^3^ Chinese Academy of Inspection & Quarantine Greater Bay Area Zhongshan Guangdong People's Republic of China; ^4^ Tianjin Key Laboratory of Food Biotechnology, School of Biotechnology and Food Science Tianjin University of Commerce Tianjin China

**Keywords:** donkey oil, incisional wound damage, transcriptome, wound healing

## Abstract

**Background:**

The identification of cosmetic ingredients that promote wound healing with minimal inflammation is urgently needed. Donkey oil (DO) is a promising candidate due to its nutritional richness and biosafety.

**Aims:**

This study aimed to evaluate the efficacy of DO in promoting wound healing and to elucidate its underlying mechanisms of action.

**Methods:**

A murine incisional wound model was established. Wounds were treated with 12.5%, 25%, or 50% DO. Healing was assessed through phenotypic observation, H&E staining, quantification of inflammatory cytokines (IL‐1α, IL‐6, PGE2, and CCL2) and VEGF, transcriptome sequencing, and qPCR validation.

**Results:**

Treatment with 25% and 50% DO significantly accelerated wound closure by 35.22% and 34.33%, respectively, and reduced inflammatory cell infiltration by 63.77% and 79.00%. Furthermore, DO preserved skin structural integrity, concurrently inhibited inflammatory cytokines increased VEGF levels, and reduced MMP‐9. To explore the potential mechanism, transcriptome analysis identified 13 603 shared differentially expressed genes (DEGs). Subsequent GO and KEGG enrichment analyses indicated these DEGs were involved in key processes like actin cytoskeleton organization and pathways related to anti‐infection and cell repair. Finally, qPCR confirmed the reversal of IL‐6 and CCL2 expression and the upregulation of TGF‐α.

**Conclusions:**

DO significantly promotes wound healing by modulating anti‐inflammatory responses and enhancing tissue repair mechanisms, demonstrating its great potential as an effective cosmetic ingredient.

## 1 Introduction

As the largest human organ, the skin protects the human body from external damage and microbial invasion, maintains homeostasis, and prevents the loss of body fluids, electrolytes, and nutrients [1]. An estimated 4511 operations per 100 000 population are carried out annually worldwide, equating to one operation each year for every 22 people [2]. This figure is higher in high‐income countries. Incisions are wounds caused by sharp objects, such as intentional or unintentional cuts during surgery. It is well known that wound healing is a complicated and dynamic process. Hemostasis, inflammation, proliferation, and tissue remodeling are four basic processes of wound healing [3]. These four phases overlap considerably and are supported by different mediators including immune cells, fibroblasts, pro‐ and anti‐inflammatory cytokines, and growth factors that contribute to new tissue formation [4]. Skin wound therapy may be categorized into two main groups: conventional procedures and regenerative approaches. Traditional methods of treating wounds often include measures to limit infection, regular changes of dressings, and the removal of wrecked tissues by debridement [5]. Regenerative wound healing encompasses a range of developing biomedical technologies, such as bioactive biomaterials, innovative dressings for wounds, treatment with stem cells, growth factor administration, gene therapies, and bioengineered skin grafts [6]. However, the restricted bioavailability of wound healing drugs like growth factors or cytokeratin, caused by their quick elimination from the wound site, imposes limitations on their practical use. Until now, it is urgent and has broad application prospects to find the potential natural products or ingredients in promoting skin wound healing.

Facing the challenge of wound healing in medical esthetics and minimally invasive procedures, exploring natural substances with enhanced wound‐repairing efficacy is of great importance. Many natural products have been reported to be efficacious and safe against skin inflammation during wound healing [7], such as 7, 8‐dimethoxycoumarin (DMC) [8], curcumin [9], *Ficus carica* extraction and *Ziziphus jujuba* leaves extraction [10]. The above natural products are effective in relieving skin inflammation through various mechanisms such as NF‐κB inhibition, COX‐2 downregulation and antioxidant activity. Among them, some natural plant oils, such as olive oil, sunflower seed oil, coconut oil et al., and animal oils, such as snake oil and horse oil, have been extensively reported to repair the skin barrier, provide antibacterial components, aid in wound healing, prevent skin aging and possess anti‐inflammatory properties [11, 12, 13]. Compared with lard, beef tallow, and mutton tallow, the disclosure of the donkey oil (DO) component indicates superior higher levels of unsaturated fatty acids (UFAs) and essential fatty acids, such as oleic acid (32.30%), linoleic acid (12.90%), and palmitic acid (26.33%), along with a low content of saturated fatty acids [14]. Especially, DO contains *n*‐3 and *n*‐6 polyunsaturated fatty acids (PUFAs), which have been reported to promote the process of neovascularization, extracellular remodeling, migration, and cellular differentiation of skin cells during the skin healing process [15, 16, 17]. Besides that, DO contains a high concentration of vitamin E, acting as an essential regulator for skin immune function and metabolic processes, which has been revealed to protect skin from lipid peroxidation, photoaging and UV‐induced immunosuppression [18, 19]. In summary, DO holds considerable potential in the fields of medicine and healthcare together with its edible biosafety. However, few studies have focused on its exact skin wound healing function and quantified its efficiency and capability.

In this study, a murine incisional wound model was established to investigate the healing function of DO on skin wounds, Three doses of DO were applied in the healing skin wound model and their effects on inflammatory factors and wound‐repair were studied. Importantly, all the differentially expressed genes (DEGs) and related pathways in response to DO under incisional wound conditions were systematically analyzed based on the transcriptome sequencing, providing a theoretical and data‐driven foundation for its application in the cosmetic industry.

## 2 Materials and Methods

### 2.1 Experimental Animals and Materials

The experimental animals used in this study were fifty healthy female Kunming mice, aged between 6 and 7 weeks, with weight from 18 to 25 g, which were purchased from Sbefu Biotechnology (Co. Ltd., License No.:SCXK2019‐0010, Beijing, China). Mice were adaptively fed for 1 week prior to the experimental process with room temperature at 20°C–25°C and air humidity at 50%–60%. During this period, they were fed with ordinary maintenance food and water; their physiological state was timely monitored.

### 2.2 Preparation of DO Samples

DO used in this study was provided by Dong‐E E‐Jiao Co. Ltd., Shandong, China. In our previous studies [20], we obtained the composition of DO, which contains 16 types of amino acids and their derivatives, 11 types of organic acids, 46 types of lipids, as well as carbohydrates, vitamins, and other components. DO contains significantly higher levels of unsaturated fatty acids (UFAs) and essential fatty acids, including oleic acid (32.3%), linoleic acid (12.90%), palmitic acid (26.33%), and vitamin E (8.59%).

DO stored at 4°C was mildly taken out and heated to melt into liquid form, followed by ultrasonic preparation of DO emulsions with mass fractions of 12.5% (low‐dose, LC), 25% (medium‐dose, MC), and 50% (high‐dose, HC) using sterile water. The DO samples with various concentrations were ultrasonicated until forming a homogenous oil–water emulsion without obvious separation.

### 2.3 Mice Grouping and Incisional Wound Model Establishment

Fifty Kunming mice were randomly divided into five groups (*N* = 10 each per treatment), including the blank group (BC), the model group (NC), 12.5% DO group (LC), 25% DO group (MC), and 50% DO group (HC). Except for the BC group, the remaining groups were subjected to incisional wound modeling.

Before modeling, the hair on the back of mice was shaved by a shaver with an area of 3 cm × 3 cm; then the animal depilatory cream was applied to the shaved area for 3 min. The depilatory cream and the residual hair were wiped off with a cotton ball containing 75% alcohol. After further disinfection with iodophor, the mice were anesthetized with diethyl ether and the 12 mm long full‐thickness longitudinal incisions were created on the shaved dorsal area [21].

**FIGURE 1:**
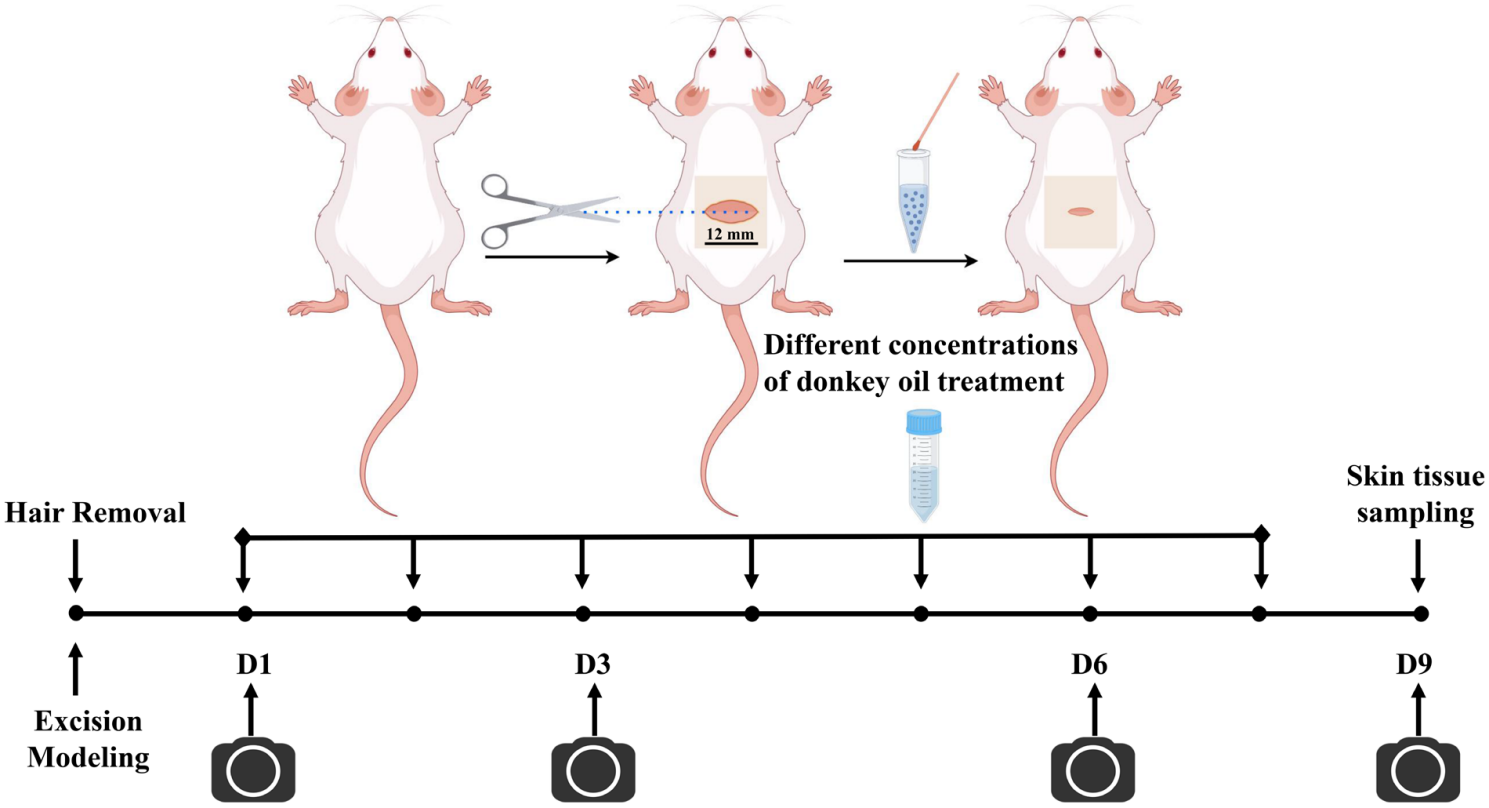
Schematic diagram of modeling process for a murine skin incisional model.

After the establishment of the wound model, different concentrations of DO samples were smeared to heal the wound respectively. Treatments were applied since 15 min after injury, and the mice were sacrificed on the 9th day. The wound healing conditions were captured on Day 1, Day 3, Day 6, and Day 9 (Figure 1). The wound area was determined using ImageJ software and the wound closure rate was calculated by Equation (1):

(1) $$\text{Wound Closure Rate}\%=\left(\left({\text{Area}}_{0}-{\text{Area}}_{d}\right)/{\text{Area}}_{0}\right)\times 100\%$$

where “d” represents the day, like 3rd, 6th, and 9th day.

### 2.4 Histological Analysis

The wounded tissue was collected on the 9th day after treatment, and fixed with 4% paraformaldehyde solution for 24 h. The tissue was then processed, embedded in paraffin, and sliced into 4 μm sections using a microtome. Skin sections were stained with hematoxylin and eosin (H&E) for histopathological analysis [22, 23].

### 2.5 Measurement of Cytokines Related to Inflammatory Response

Mice skin tissue was precisely weighed and cut to 0.1 g, then mixed with nine volumes of cold phosphate buffer at a pH of 7.4. Using a grinder, the skin tissue was thoroughly homogenized on ice and subsequently centrifuged at 12 000 g for 10 min. The supernatant was carefully collected and divided into centrifuge tubes. One aliquot of the supernatant was stored at 4°C for immediate testing; the remainder was stored at −80°C for future use. Each aliquot of supernatant was tested as promptly as possible to avoid repeated freeze–thaw cycles. After preparing the homogenate of mouse skin tissue, the contents of cytokines related to inflammatory response (IL‐1α, IL‐6, PGE2, VEGF, and MMP‐9) in the dorsal back skin tissue were measured using mouse ELISA kits, following the manufacturer's protocol. The ELISA kits IL‐1α and VEGF were purchased from ProteinTech (Wuhan, China, Cat No: KE10024 and KE10009); IL‐6 from Multi Sciences (Hangzhou, China, Cat No: EK206/3‐96); PGE2 from Lanpaibio (Shanghai, China, Cat No: LP‐M05161); MMP‐9 from CUSABIO (Wuhan, China, Cat No: CSB‐E08007m).

### 2.6 RNA Extraction and Transcriptome Sequencing

The total RNA was extracted with an RNA extraction kit and RNA integrity was detected by the Agilent 2100 bioanalyzer precisely. After confirming the quality of the total RNA samples, mRNA was enriched from the total RNA using Oligo dT magnetic beads, and the libraries were constructed according to strand‐specific library construction [24]. After the library construction was completed, Qubit2.0 Fluorometer was used for preliminary quantification, and then the Agilent 2100 bioanalyzer was used to detect the insert size of the library. After the insert size met the expectation, qRT‐PCR was used to accurately quantify the effective concentration of the library to ensure the quality of the library. After raw data filtering, sequencing error rate checking, and GC content distribution checking, clean reads for use were obtained. RNA‐seq was performed on nine skin samples by Novozymes Technology Co. Ltd, Beijing, China.

### 2.7 Differential Expression Analysis and Functional Enrichment

After obtained the clean reads, the reference genomes were downloaded from the NCBI database and aligned with the Tophat software. The DESeq2R software [25] was used to screen for differential expression of genes, and differential genes were defined as |log_2_(FoldChange)| ≥ 1 and *p*adj ≤ 0.05. In addition, GO (gene ontology) function annotation and KEGG (Kyoto Encyclopedia of Genes and Genomes) pathway enrichment were analyzed through lusterProfiler software of the differential gene sets. The screening criteria for significantly enriched GO and KEGG by DEGS were Bonferroni corrected pvalues of less than or equal to 0.05.

### 2.8 RT‐qPCR

RNA was extracted from mouse skin tissue using the E.Z.N.A. Total RNA Kit II (OMEGA, Norcross, Georgia, Cat No: R6934‐01). cDNA was synthesized using the All‐In‐One 5× RT MasterMix kit (ABM, Viking Way, Canada, Cat No: G592), and qPCR was performed using the BlasTaq 2× qPCR MasterMix kit (ABM, Viking Way, Canada, Cat No: G891). The qPCR primers used in this study were listed in Table 1.

**TABLE 1:** qPCR primers.

Gene	Prime	Oligonucleotide sequence (5′→3′)
IL‐6	Forward	CCACTTCACAAGTCGGAGGCTTA
	Reverse	CCAGTTTGGTAGCATCCATCATTTC
CCL2	Forward	GCTGACCCCAAGAAGGAATG
	Reverse	TGAGGTGGTTGTGGAAAAGG
TGF‐α	Forward	CCAGCATGTGTTGGTCTGAAG
	Reverse	TTGTGCACTGAGGGGGAAGG
β‐actin	Forward	GTGCTATGTTGCTCTAGACTTCG
	Reverse	ATGCCACAGGATTCCATACC

### 2.9 Statistical Analysis

The data presented in this study were depicted as the mean ± SD. One‐way ANOVA was used to statistically evaluate the data and the Tukey test was used to examine the differences between the control group and other groups. Statistical analysis and mapping were performed using SPSS 20.0 (SPSS PASW Statistic 23.0, SPSS Inc., Chicago, IL, USA) and GraphPad Prism (GraphPad Software Inc., San Diego, CA, USA). Statistical significance: *p < 0.05; **p < 0.01; ***p < 0.001.

## 3 Results

### 3.1 Phenotypes of DO on Healing the Murine Incisional Wounds

**FIGURE 2:**
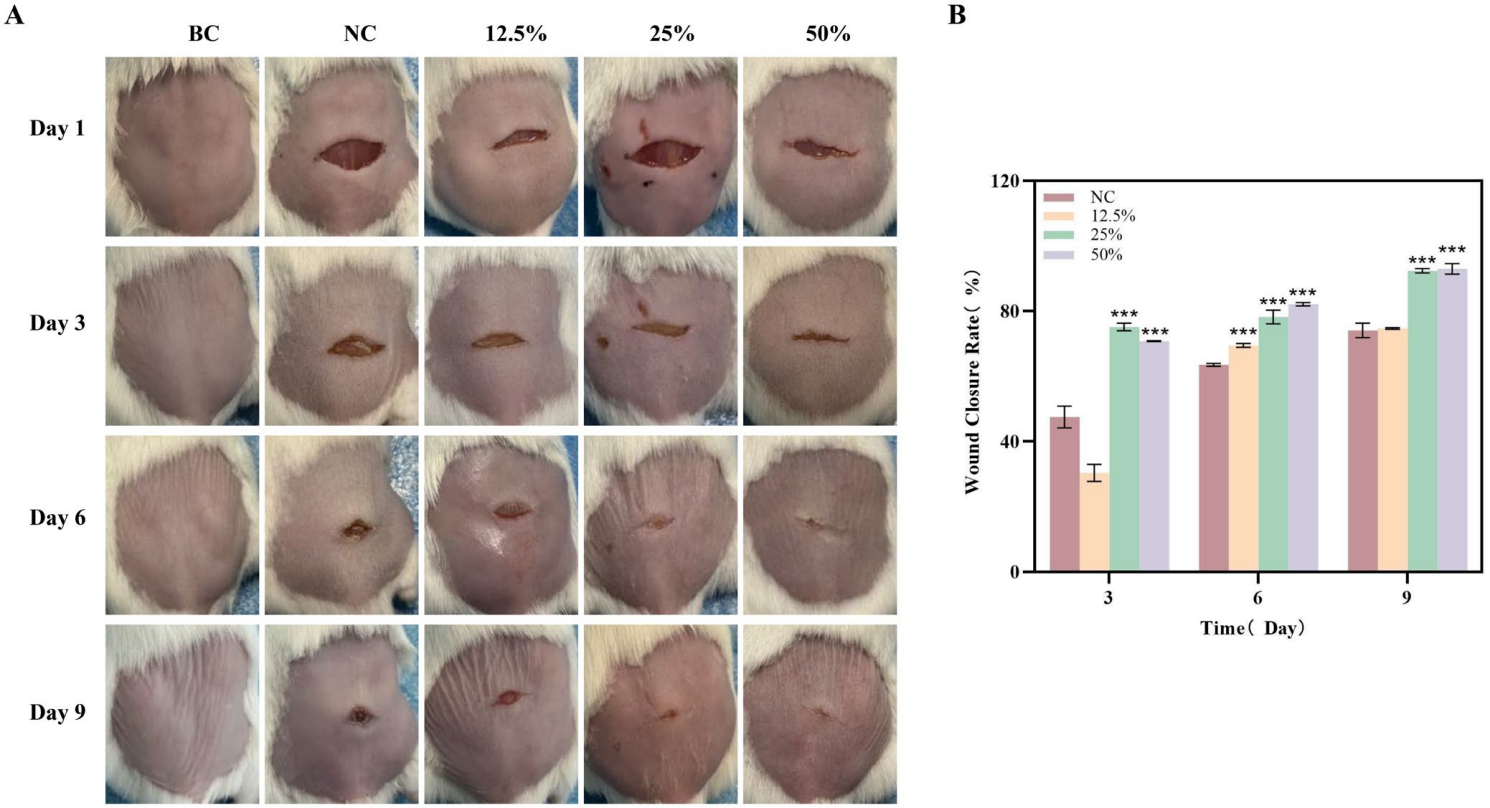
(A) Phenotypes of different treatment groups on healing the incisional wounds. (B) The wound closure rate of different treatment groups. (BC: Blank group, NC: Model group, 12.5%: Low‐dose group, 25%: Medium‐dose group, 50%: High‐dose group, *p < 0.05; **p < 0.01; ***p < 0.001 and *n* = 3. The wound area was determined using ImageJ software).

In order to figure out the roles of DO on healing the murine wounded skin, we established the murine incisional wound model. The wound healing photographs were captured on days 1, 3, 6, and 9 after injuring and the healing effects of DO could be evaluated based on the wound size and skin condition (Figure 1). As shown in Figure 2A, the incisional wound of the mice in each group gradually healed and scab marks appeared with the prolongation of time. Compared with the model group (NC), 25% DO and 50% DO both showed analogously accelerated wound healing processes on Day 3 and Day 6 than 12.5% DO. Moreover, on the 9th day after treatment, both the wound healing conditions of the 25% DO and 50% DO exhibited higher efficiency than that of the NC group and the 12.5% DO group, with a higher degree of healing, lighter scab marks, and smoother skin at the site of wounds, while the NC and 12.5% DO groups had a lower degree of wound healing and more obvious scab scars. By measuring the wound closure rate using ImageJ software, our results indicated that the wound closure rates of mice in the 25% and 50% DO groups were significantly higher than those in the model group on Days 3, 6, and 9 (*p* < 0.001) as shown in Figure 2B. To sum up, the 25% and 50% DO could heal the incisional wounded tissue effectively.

### 3.2 Histological Analysis of DO on Healing the Murine Incisional Wounds

**FIGURE 3:**
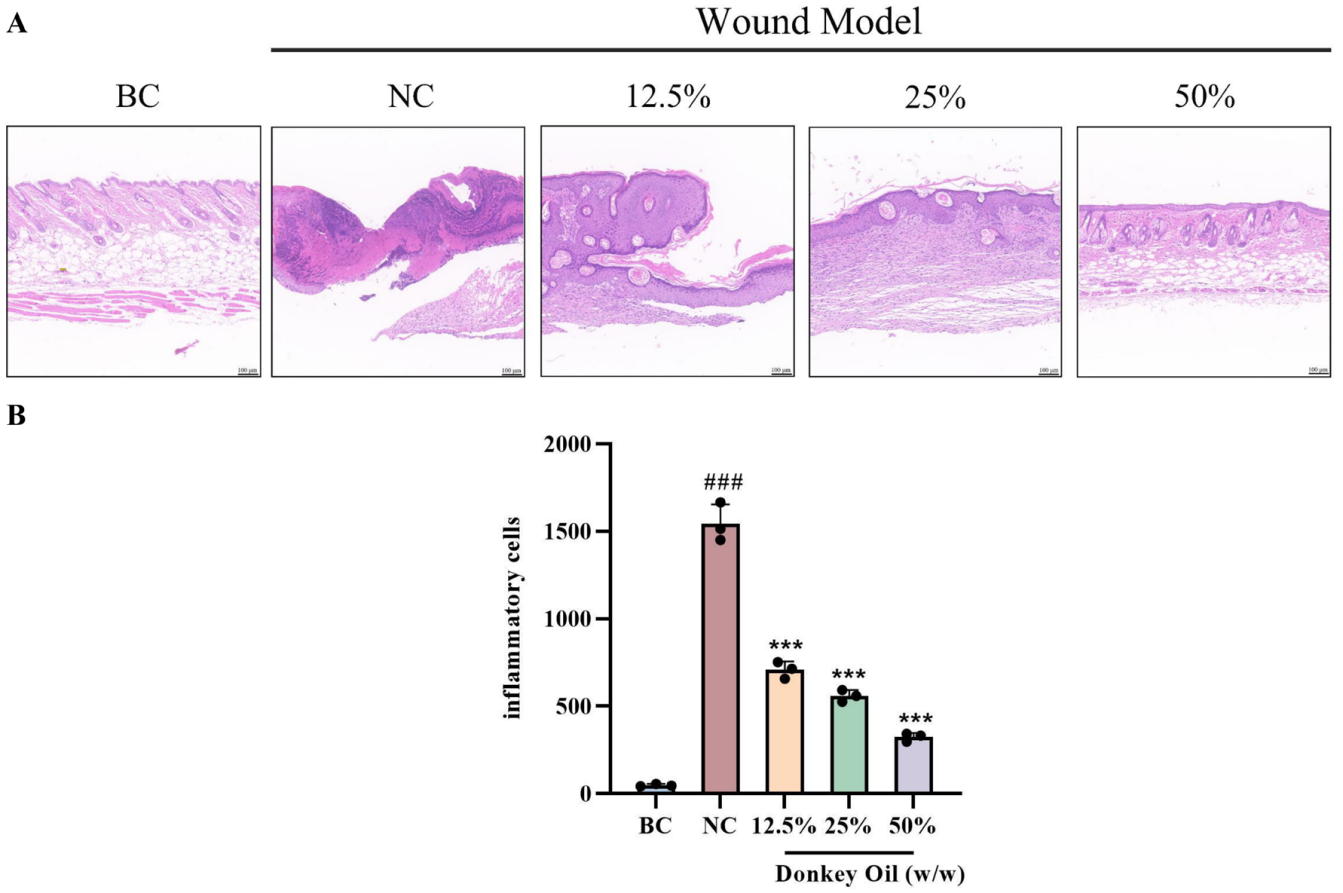
(A) Photomicrographs of skin sections of all the groups stained by H&E. (B) Counting of the inflammatory cells in all the groups. (BC: Blank group, NC: Model group, 12.5%: Low‐dose group, 25%: Medium‐dose group, 50%: High‐dose group. All images were enlarged to 200×). ^###^ *p* < 0.001 vs. BC group; ***p < 0.001 vs. NC group.

H&E staining was used to evaluate skin regeneration from a histological perspective. The healing of skin lesions in each group is shown in Figure 3A. In the BC group, the structure of the skin was complete. The epidermal layer was composed of the stratum corneum, transparent layer, granular layer, spine layer and basal layer. The dermal papilla could be observed in the dermal layer; the collagen fibers were arranged neatly, the skin appendages hair follicles and sebaceous glands were arranged in an orderly manner. Compared with the BC group, obvious wound surfaces and scabs were observed in the skin tissue of the NC group, which was incomplete with the transparent layer, granular layer, spine layer and basal layer disappearing in the epidermal layer; intact collagen fibers, skin appendages sebaceous glands and hair follicles were not seen in the dermal layer, granulation tissue appeared in the dermis but had not yet filled the wounds. Compared with the NC group, different doses of DO groups showed distinct effects on the structural repair of wounded skin tissue. Specifically, in the 12.5% DO group, the stratum corneum was complete but markedly thickened, the dermis was in the repair stage and the granulation tissue filled the wounds. In the 25% DO group, the skin structure was complete with decreased epidermal thickness and normalized stratum corneum thickness; skin appendages were visible in the dermis and collagen fibers were arranged neatly. However, a small amount of hair follicles, sebaceous glands and inflammatory infiltration was observed in the dermis. In the 50% DO group, the epidermis and dermis became intact, the thickness of the stratum corneum returned to normal, skin appendages were visible in the dermis and collagen fibers were arranged neatly. Notably, the 50% DO group exhibited a higher density of hair follicles and sebaceous glands compared to the 25% DO group, along with a significant reduction in inflammatory infiltration. The above pathological results indicated that the 50% DO group exhibited the best effect on the healing of skin wounds, followed by the 25% DO group, while the effect of the 12.5% DO group was relatively poorer. By counting the number of inflammatory cells in HE staining histology as shown in Figure 3B, we found that 12.5%, 25%, and 50% DO treatment decreased the inflammatory cells in comparison with the NC group (skin wounds), respectively, indicating the significant incision‐repair function of DO.

### 3.3 Effects of Different Concentrations of DO on the Inflammatory Factors Secreted by Injury Mice Skin Tissue

Inflammation is a natural defense mechanism of the body that involves the migration of leucocytes to the damaged tissue to destroy the inflammatory trigger or challenge [26]. A moderate inflammatory response is necessary for wound healing to remove infected and necrotic tissue and create a suitable environment for healing. If the inflammatory response is too intense or lasts too long, it can lead to chronic wounds and impede the healing process. PGE‐2 is a member of the prostaglandin family that promotes the migration and activation of inflammatory cells and enhances vascular permeability [27]. IL‐1α is a proinflammatory cytokine that stimulates the release of more inflammatory factors from other cells [28]. IL‐6 is a multifunctional cytokine involved in the regulation of immune response, inflammation and hematopoiesis [29]. In order to determine the anti‐inflammatory effects of DO during the wound healing process aside from H&E staining, the levels of these three factors were determined at 1, 3, 6, and 9 days after injuring.

**FIGURE 4:**
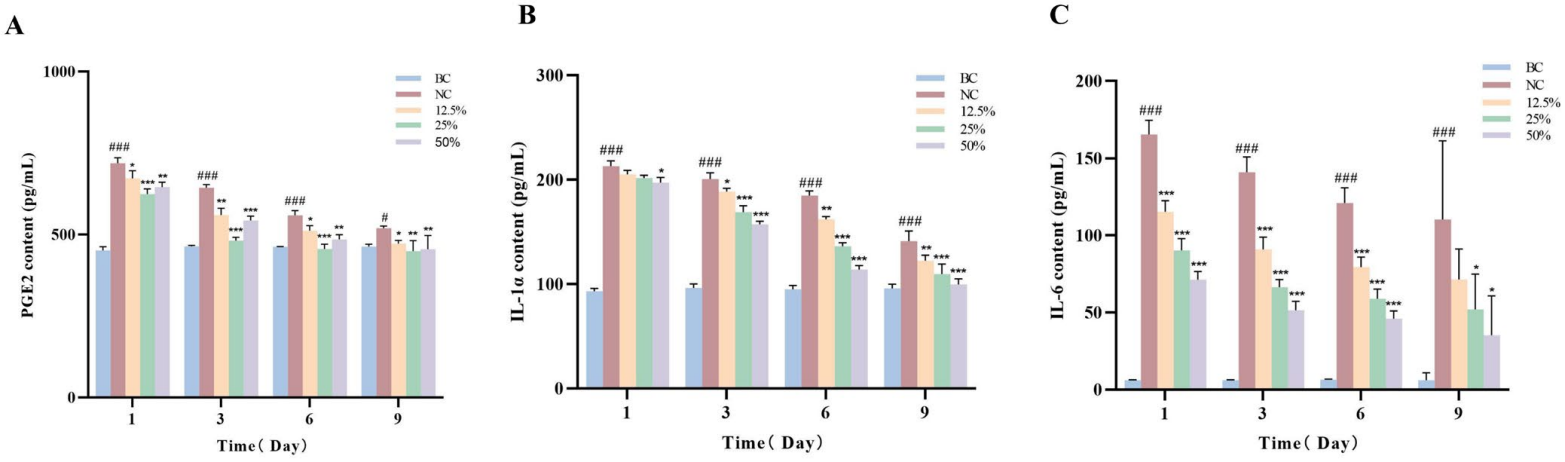
Effects of different concentrations of DO on the content of inflammatory factors PGE2 content (A), IL‐1α content (B) and IL‐6 content (C) in the skin tissue of mice after incision injury. ^###^ *p* < 0.05, ^###^ *p* < 0.001 vs. BC group; *p < 0.05, **p < 0.01, ***p < 0.001 vs. NC group.

As shown in Figure 4, compared with the BC group, whether the content of PGE2 (A) or IL‐1α (B), and IL‐6 (C) in the NC group peaked at 1 day after incision injuring and gradually decreased with the prolongation of time, indicating that there was an inflammatory reaction in the skin tissue of the mice after injury. In accordance with the results of photomicrographs (Figure 2) and H&E staining (Figure 3), there was a significant decreasing tendency of the PGE2 content in the mice skin tissue after application of the three concentrations of DO (Figure 4A). For example, in comparison with the NC group after 9 days of injury, the PGE2 content in the 12.5% group was 472.08 pg/mL, reduced by 9.06% (*p* < 0.05). The PGE2 content in the 25% group was 448.31 pg/mL, reduced by 13.64% (*p* < 0.01). The PGE2 content in the 50% group was 454.93 pg/mL, reduced by 12.37% (*p* < 0.01). These results showed that all three DO groups could reduce PGE2 content induced by the inflammatory response in mice.

Analogous to the results of PGE2, there was a significant trend of decrease in the IL‐1α and IL‐6 content of the skin tissue after application of DO (Figure 4B). For example, compared with the NC group at 9 days after injuring, DO treatment decreased the IL‐1α content in the 12.5% group with a percentage of 13.31% (*p* < 0.01) and decreased the IL‐6 content with a percentage of 35.26%, respectively. More apparent than the effect of 12.5% DO, the 25% group reduced the IL‐1α content by 22.44% (*p* < 0.001) and reduced the IL‐6 content by 52.90% (*p* < 0.05), respectively. With the best effect, the 50% group obviously reduced the IL‐1α content by 29.36% (*p* < 0.001) and reduced the IL‐6 content by 68.01% (*p* < 0.05) compared with the NC group. The results showed that DO could inhibit the wound‐induced production of PGE2, IL‐1α and IL‐6 in a dose‐dependent manner and 25% and 50% DO showed an optimum regulatory effect on PGE2, IL‐1α, and IL‐6 in mice, respectively, facilitating the wound healing by decreasing the levels of PGE2, IL‐1α and IL‐6 after wound treatment.

### 3.4 Effects of Different Concentrations of DO on VEGF and MMP‐9 Factors Secreted by Injury Mice Skin Tissue

Vascular endothelial growth factor (VEGF), a signaling protein produced by cells, stimulates angiogenesis, plays a role in normal physiological functions such as bone formation, hematopoiesis, wound healing and development. During the early stages of wound healing, extensive new blood vessels (granulation tissue) are required to supply nutrients and oxygen to repair cells such as fibroblasts and keratinocytes; VEGF plays a crucial role in this process. Matrix metalloproteinase‐9 (MMP‐9) is one of the most investigated and studied biomarkers of the MMPs family. It has been proved that when the body is in an inflammatory state, activated leukocytes could secrete large amounts of MMP‐9 to all tissues and organs throughout the body [30, 31].

**FIGURE 5:**
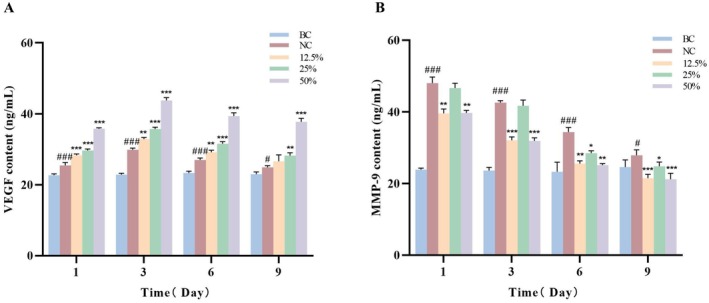
Effects of different concentrations of DO on the content of VEGF (A) and MMP‐9 (B) in the skin tissue of mice after incision injury. ^#^ *p* < 0.05, ^###^ *p* < 0.001 vs. BC group; *p < 0.05, **p < 0.01, ***p < 0.001 vs. NC group.

Figure 5A illustrates the dynamic changes in VEGF levels during wound healing. Compared with the BC group, the NC group exhibited a rapid increase in VEGF content in skin tissue post‐incision, peaked at 29.88 ng/mL at 3 days after incision injuring and gradually decreased with the prolongation of time. DO treatment further elevated VEGF content. For instance, in comparison with the NC group after 9 days of injuring, the 25% group exhibited a 13.39% increase in VEGF content (*p* < 0.01). Compared to the 25% donkey oil group, the 50% donkey oil group showed a further significant increase of 51.35% (*p* < 0.001).

The dynamic changes in MMP‐9 content are illustrated in Figure 5B. Following the incision, MMP‐9 content in mouse skin tissue rapidly increased compared with the BC group, in order to clear damaged matrix, promote cell migration, and regulate growth factors, and peaked at 1 day after incision injuring. Subsequently, MMP‐9 content gradually decreased over time to return to normal. MMP‐9 content in mouse skin tissue treated with the three concentrations of DO showed a decreasing trend. For example, compared with the NC group after 9 days of injuring, in the 12.5% and 50% DO groups, the MMP‐9 content was significantly reduced by 22.89% and 23.93% (*p* < 0.001) respectively. In the 25% group, the MMP‐9 content was significantly reduced by 11.02% (*p* < 0.05). These findings indicated DO accelerates skin wound healing through a dual mechanism: promoting angiogenesis and inhibiting excessive degradation of the extracellular matrix.

### 3.5 Transcriptomic Analysis Reveals the Regulatory Mechanism of DO in Incisional Wounds Healing

From the above aspects of wound healing phenotypes as well as anti‐inflammatory and skin tissue growth promotion, our present results showed the 50% DO an optimum concentration for incision‐repair. In order to further reveal the possible regulatory mechanism of DO in wounded tissue repair, we selected the Blank group (BC), the Incision model group (NC) and the High‐dose DO group (HC) to perform transcriptomics analysis in an attempt to reveal the repair mechanism by exploring the signal pathways that may be affected by DO.

**FIGURE 6:**
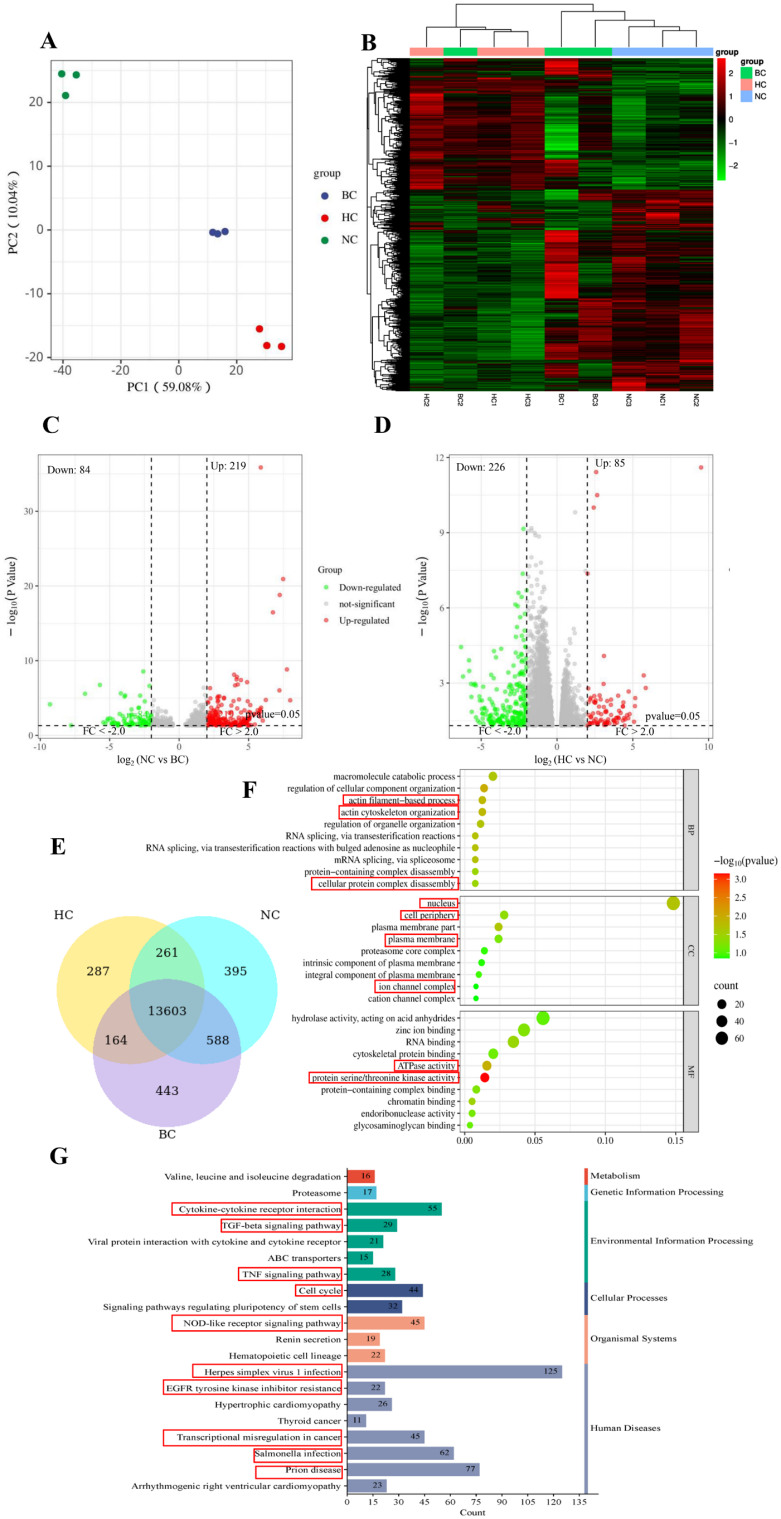
Transcriptome results indicated the role of donkey oil in accelerating the healing of incision wounds by regulating inflammatory responses and promoting tissue remodeling. (A) PCA plot illustrating distinct transcriptomic profiles of mouse skin tissue among Blank group Incision model group and High‐dose DO groups. (B) Heatmap displaying DEGs among the three experimental groups. (C) Volcano plots of DEGs between Incision model group vs. Blank group. (D) Volcano plots of DEGs between High‐dose DO group vs. Incision modelgroup. (E) Venn diagram showing overlapping DEGs between Incision model group, Blank group and High‐dose DO group. (F) GO enrichment analysis of DEGs across biological process, cellular component, and molecular function categories. (G) KEGG pathway enrichment analysis of DEGs. Dot size corresponds to the number of genes, and color represents the significance level.

Principal component analysis (PCA) revealed distinct separation among these groups, confirming the reproducibility of the experimental conditions and notable transcriptomic divergence due to incision molding and DO treatment (Figure 6A). DEGs were identified by comparing Incision model group versus Blank group, as well as the High‐dose DO group. A heatmap illustrated expression patterns of the top identified DEGs, highlighting clear differences between the Blank group and Incision model groups, with High‐dose DO treatment partially reversing these transcriptomic alterations (Figure 6B). The volcano plots further visualized these gene expression changes, clearly displaying significant DEGs induced by incision modeling and subsequently modulated by DO treatment (Figure 6C,D). A Venn diagram analysis identified 13 603 DEGs shared between Incision model group vs. Blank group and High‐dose DO group vs. Incision model group, suggesting these genes were specifically modulated by DO intervention in response to incision‐induced damage (Figure 6E). These 13 603 overlapping genes were used for subsequent analyses. Gene Ontology (GO) enrichment analysis showed that these DEGs were significantly involved in actin filament‐based process, actin cytoskeleton organization, cellular protein complex disassembly, nucleus, cell periphery, plasma membrane, ion channel complex, ATPase activity, and protein serine/threonine kinase activity (Figure 6F). KEGG pathway enrichment further indicated significant involvement in critical signaling pathways, among the top 10 enriched pathways including Herpes simplex virus 1 infection, Prion disease, *Salmonella* infection, transcriptional misregulation in cancers, cytokine–cytokine receptor interaction, TNF signaling pathway, EGFR tyrosine kinase inhibitor resistance, NOD‐like receptor signaling pathway, cell cycle, TGF‐beta signaling pathway, et al. (Figure 6G), highlighting that DO promoted the healing of incised wounds by preventing the inflammatory responses and playing its anti‐infection function on one hand, and by facilitating the proliferation and migration of fibroblasts and keratinocytes to enable tissue remodeling by regulating cytokine–cytokine receptor interaction, TNF signaling pathway, NOD‐like receptor signaling pathway and TGF‐beta signaling pathway, et al. Collectively, these transcriptomic data strongly support the hypothesis that DO promoted the anti‐inflammatory responses of the wounded mouse exemplified by the top three gene enriched pathways including Herpes simplex virus 1 infection, Prion disease, *Salmonella* infection pathways, which were all related to anti‐infection responses after virus or bacterial invasion. Meanwhile, Cytokine, EGFR, cell cycle, TGF‐beta signaling pathway were all related to the repair and regeneration of cells upon wounding, implicating the damage‐repair function of DO.

### 3.6 RT‐qPCR Detection of Differentially Expressed Genes by Transcriptomic Analysis

**FIGURE 7:**
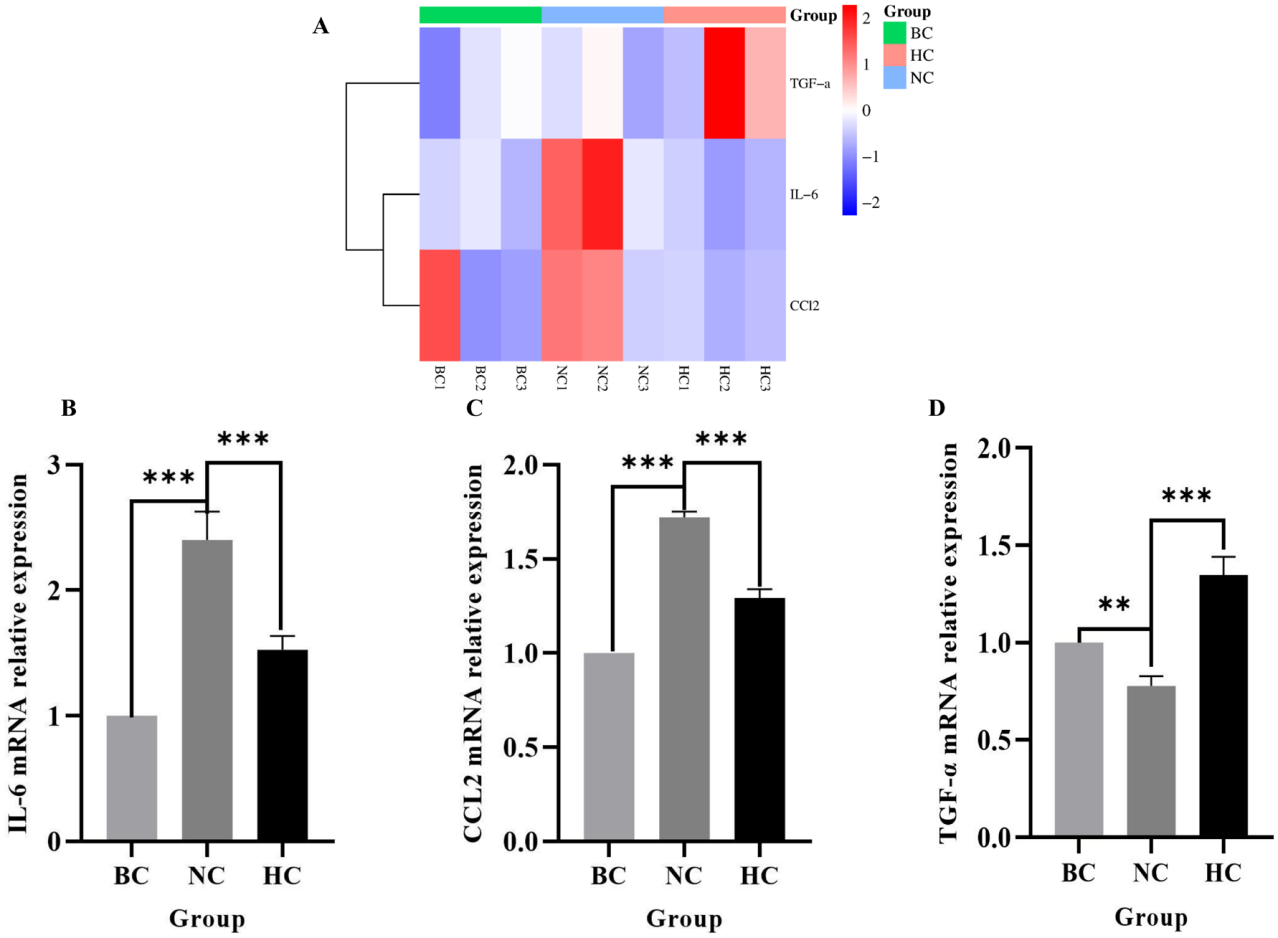
(A) Heatmap of *IL‐6*, *CCL2*, and *TGF‐α* Expression. (B–D) *IL‐6*,*TGF‐α*and *CCL2* mRNA relative expression in different groups. **p < 0.01; ***p < 0.001.

To testify to the validity of the transcriptome analysis and gene differential expression, we selected key genes from DEGs identified via RNA sequencing and quantified their mRNA expressional levels by RT‐qPCR. Meanwhile, to detect the role of DO in anti‐inflammation and wound injury repair promotion functions, we detected the transcriptional expression of IL‐6 and CCL2, which were chosen due to their pivotal cytokines in inflammatory responses, while TGF‐α—a member of the epidermal growth factor family primarily driving reepithelialization. By analyzing the relative expression of IL‐6, CCL2, TGF‐α based on the transcriptomic sequencing as shown in Figure 7A, incision injury obviously increased the expression of IL‐6 and CCL2, but dramatically decreased the expression of TGF‐α compared with the BC group. Inversely, the DO treatment group significantly reversed these phenomena. Interestingly, the transcriptional level of TGF‐α was dominantly higher than that of the BC group. Consistent with transcriptome data, by qPCR validation as shown in Figure 7B,C, incision treatment significantly upregulated IL‐6 and CCL2 gene expression (*p* < 0.001), while DO treatment markedly reversed the upregulation of these two inflammatory genes (*p* < 0.001). Simultaneously, the incision induced a significant downregulation of TGF‐α expression (*p* < 0.001), while DO treatment obviously withdrew the responses by incision injury and even reached a higher level than the BC group (Figure 7D), suggesting the potent induction of TGF‐α expression by DO. These findings indicated that DO effectively modulated the expression of inflammatory factors post‐incision and upregulated epidermal growth factor family genes, thereby promoting tissue repair.

## 4 Discussion

In this study, we investigated the effects of three different concentrations of DO on wound healing in a murine incisional wound model. Phenotyping and HE staining showed that the 25% and 50% DO groups had better effects on wound healing, By analyzing the changes in inflammatory factors (IL‐1α, IL‐6, and PGE2) and tissue repair‐related factors (VEGF, MMP‐9) in skin tissue, we proved the healing effects of DO from the aspect of anti‐inflammation repair and tissue regeneration. Furthermore, the molecular mechanism of DO in wound healing was analyzed through transcriptome sequencing. The top 10 enriched pathways included *Herpes simplex virus 1 infection, Prion disease, Salmonella* infection, transcriptional misregulation in cancers, cytokine–cytokine receptor interaction, TNF signaling pathway, EGFR tyrosine kinase inhibitor resistance, NOD‐like receptor signaling pathway, Cell cycle, TGF‐beta signaling pathway, et al. It could be seen that the differential genes in response to DO mainly enriched two types of pathways: virus and bacterial infection related pathway, and cell regeneration and repair signaling pathway, implicating the function of DO in wound healing.

In order to address the anti‐inflammatory and wound‐repair role of DO, we firstly elucidated the composition of DO. As described in our previous study, DO contains 16 types of amino acids and their derivatives, 11 types of organic acids, 46 types of lipids, as well as carbohydrates, vitamins, and other components. Especially, the levels of unsaturated fatty acids (UFAs) and essential fatty acids such as oleic acid reached 32.3%, linoleic acid reached 12.90%, palmitic acid reached 26.33%, and vitamin E reached 8.59%. Additionally, by the ultrasonic‐assisted solvent extraction method, DO has a relative density of 0.908, a moisture content of 0.68%, an acid value (KOH) of 0.58 mg/g, a peroxide value of 0.018 g/100 g, and an iodine value (I) of 85 g/100 g. These results indicate that DO has a low content of free fatty acids and a higher content of unsaturated fatty acids compared with lard, beef tallow, and mutton tallow, thus possessing high economic value [20].

As reported, oleic acid, linoleic acid, palmitic acid, and vitamin E play crucial roles in skin repair and anti‐inflammatory processes. Oleic acid enhances the penetration of active ingredients, suppresses the expression of inflammatory factors such as NO and IL‐6, and boosts antimicrobial activity [32], topical application of 3% oleic acid effectively alleviates UVB‐induced skin pain and inflammation [33]. Linoleic acid synergistically regulates stratum corneum lipid structure with oleic acid, activating PPAR‐α receptors to promote ceramide synthesis [34]. As a key component of skin barrier lipids, palmitic acid can accelerate wound healing via an anti‐inflammatory effect, especially in the stages of granulation tissue formation and remodeling [35]. Vitamin E, with its potent antioxidant capacity, scavenges free radicals, suppresses inflammation, and promotes collagen synthesis and tissue regeneration [26]. In our present research, we found that incision in skin stimulates the secretion of inflammatory mediators (PGE2, IL‐6, IL‐1α) in skin tissue. DO treatment reduces the overexpression of these inflammatory mediators after injuring, preventing excessive inflammatory responses and thereby accelerating wound healing (Figure 4). Additionally, DO promotes wound healing by stimulating angiogenesis and inhibiting extracellular matrix degradation through elevating VEGF levels and reducing MMP‐9 levels in skin tissue (Figure 5).

**FIGURE 8:**
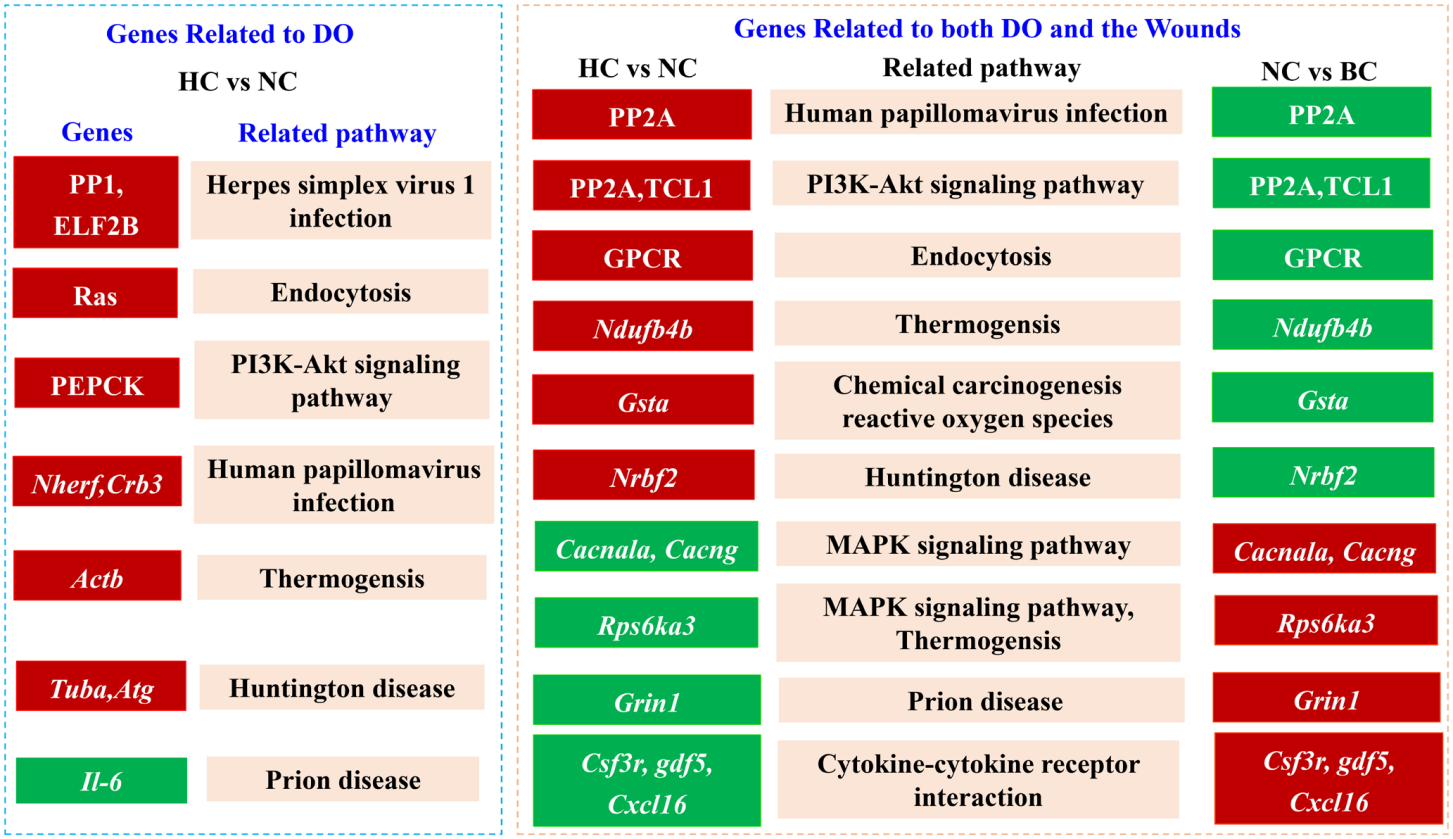
Clarification of core genes in co‐regulated pathways between HC vs. NC and NC vs. BC groups. Red represents upregulated, green represents downregulated.

Furthermore, through transcriptome analysis of the HC and NC groups, 3409 differential genes were screened, among which 2049 genes were upregulated and 1360 genes were downregulated. Through comprehensive analysis of the representative genes in the top ten pathways, these genes could be classified into two kinds including genes related to DO and genes related to both the wound and DO (Figure 8). Genes upregulated/downregulated in the HC vs. NC group showed a different expression tendency in the NC vs. BC group, indicating the specific and important function of DO in wound healing. For example, *PP2A*, *TCL1*, and *Ndufb4b* were upregulated in the HC vs. NC group, whereas both genes were downregulated in the NC vs. BC group (Figures S1 and S2); also the HC vs. NC group resulted in a significant downregulation of *Grin1* and *Rps6ka3*, which reversed the upregulation of these genes in the NC vs. BC group (Figures S3 and S4). For example, among these genes and pathways, *PP2A* plays important roles in energy metabolism, DNA damage and repair, protein translation, cell cycle regulation, and signal transduction by catalyzing protein dephosphorylation and participating in the regulation of many enzymes and transcription factors [27], *Rps6ka3* promotes protein synthesis by phosphorylating downstream target proteins to provide a material basis for cell growth and proliferation [28, 29]. The PI3K‐Akt signaling pathway has been reported to be related to wound healing, which was in accord with our results and highlighted the function of DO [30].

In a viewpoint to explain the potential mechanism of DO on wound healing, we strongly deduced that the function of DO on anti‐inflammation and wound healing function was closely related to its abundance presentation of oleic acid, linoleic acid, palmitic acid, and vitamin E, as well as its low concentration of PUFAs ingredients, particularly *n*‐3 PUFAs (EPA and DHA), which serve as essential precursors for anti‐inflammatory and pro‐resolution mediators, facilitating wound healing through multiple mechanisms including anti‐inflammation, promotion of cell regeneration, modulation of immune responses, reduction of systemic inflammatory responses, decreasing infection risks, and accelerating recovery [31]. DHA activates the GPR120 receptor in skin tissue, thereby hastening wound healing and diminishing inflammatory factor expression [36]. Additionally, the collaborative participation of the ingredients, that were in wide variety but in low proportion, such as amino acids and its derivatives, organic acids and other trace elements, could not be excluded from the wound healing function exerted by DO.

Our present study has several strengths, including the macroscopic observation of phenotype accomplished by DO in a mouse incision wound model and the microscopic molecular mechanism digging including both the measuring of inflammatory factors, VEGF, MMP‐9, and the transcriptome sequencing analysis, by which solid evidences were supplied to support the wound healing of DO. However, one limitation of our study is that the candidate specific genes that robustly respond to DO treatment had not been precisely figured out based on our present results. Further investigations including the nutrition supplier or its role in skin microecology during wound healing were urgently needed to disclose more functions and mechanisms of DO.

## 5 Conclusions

Our study demonstrated that DO exhibited significant wound healing properties, as evidenced by accelerated healing rates in a murine incisional wound model. However, treatment with DO, particularly at a high dose (50%, w/w), not only enhanced the overall healing process but also reduced inflammatory cell infiltration compared with the NC group at 9 days after injury. High dose DO treatment decreased the PGE2, IL‐6 and IL‐1α content by 12.37% (*p* < 0.01), 68.01% (*p* < 0.05) and 29.36% (*p* < 0.001), respectively. It also preserved the structural integrity of the epidermis and dermis. Transcriptomic analysis highlighted the role of DO in modulating inflammatory responses and cell regeneration‐related metabolic pathways. Given the potential therapeutic properties of DO, we will focus on exploring its further application in the cosmetic industry.

## Author Contributions

Conceptualization: J.Y. and H.T. Methodology: J.C., J.H. and L.H. Software: G.L. Validation: J.Y., H.T. and L.H. Investigation: M.L. and W.Z. Data curation: J.C., G.L. and T.W. Writing – original draft preparation: J.Y. and L.H. Writing – review and editing: J.Y., H.Z. and B.H. Supervision: H.T. and W.Z. All authors have read and agreed to the published version of the manuscript.

## Ethics Statement

The animal study protocol was approved by the Institute Research Ethics Committee of Tianjin Normal University (protocol code: 2023031304, date of approval: 13 March 2023).

## Consent

The authors have nothing to report.

## Conflicts of Interest

The authors declare no conflicts of interest.

## Supporting information


**Figure S1:** The PI3K‐AKT signaling pathway in the KEGG analysis. (A) HC group vs. NC group. (B) NC group vs. BC group.
**Figure S2:** The thermogenesis pathway in the KEGG analysis. (A) HC group vs. NC group. (B) NC group vs. BC group.
**Figure S3:** The prion disease pathway in the KEGG analysis. (A) HC group vs. NC group. (B) NC group vs. BC group.
**Figure S4:** The MAPK signaling pathway in the KEGG analysis. (A) HC group vs. NC group. (B) NC group vs. BC group.

## Data Availability

The data that support the findings of this study are available from the corresponding author upon reasonable request.
