# Supplementary material for: Protective Effects of Exogenous Donkey Oil on Skin Healing Under Incisional Wound Damage
Source: J Cosmet Dermatol. 2025 Nov 18;24(11):e70550. doi: 10.1111/jocd.70550 (PMC12626390; doi:10.1111/jocd.70550)
Supplement: Supplementary file 1 — Figure S1: The PI3K‐AKT signaling pathway in the KEGG analysis. (A) HC group vs. NC group. (B) NC group vs. BC group. Figure S2: The thermogenesis pathway in the KEGG analysis. (A) HC group vs. NC group. (B) NC group vs. BC group. Figure S3: The prion disease pathway in the KEGG analysis. (A) HC group vs. NC group. (B) NC group vs. BC group. Figure S4: The MAPK signaling pathway in the KEGG analysis. (A) HC group vs. NC group. (B) NC group vs. BC group. [file JOCD-24-e70550-s001.docx]

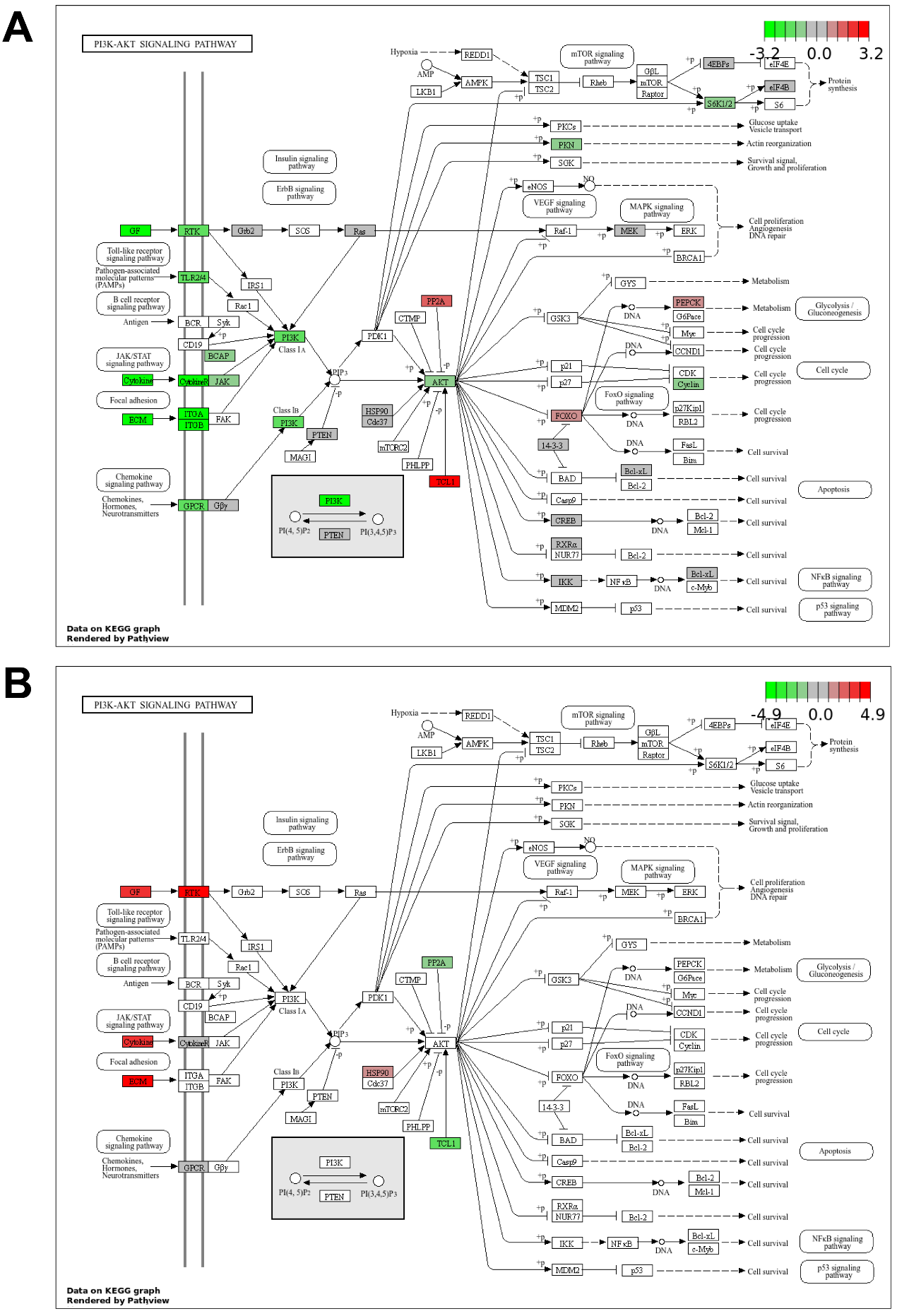


**Figure S1.** The PI3K-AKT signaling pathway in the KEGG analysis. (A) HC group vs. NC group. (B) NC group VS. BC group.


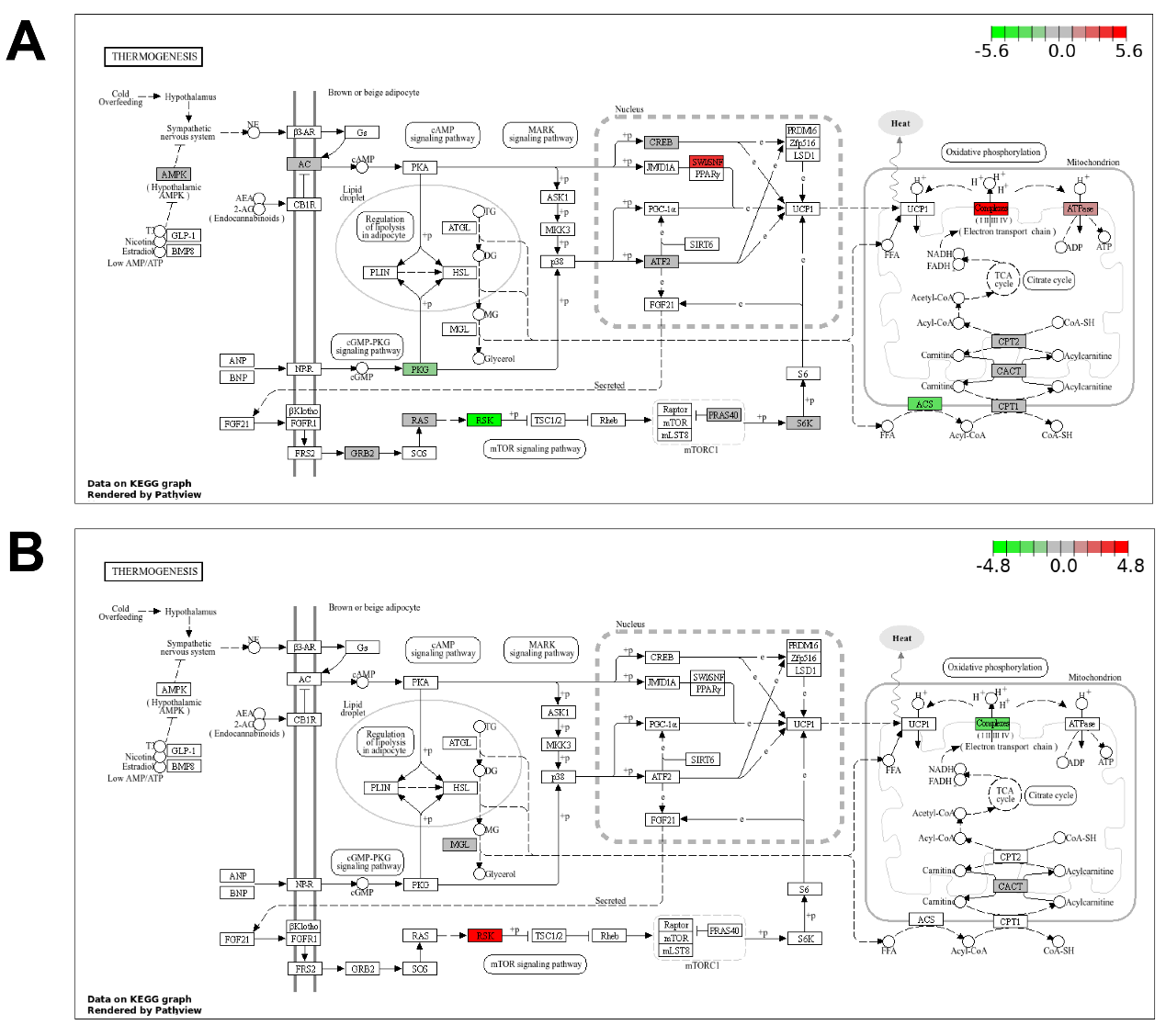


**Figure S2.** The thermogenesis pathway in the KEGG analysis. (A) HC group vs. NC group. (B) NC group VS. BC group.


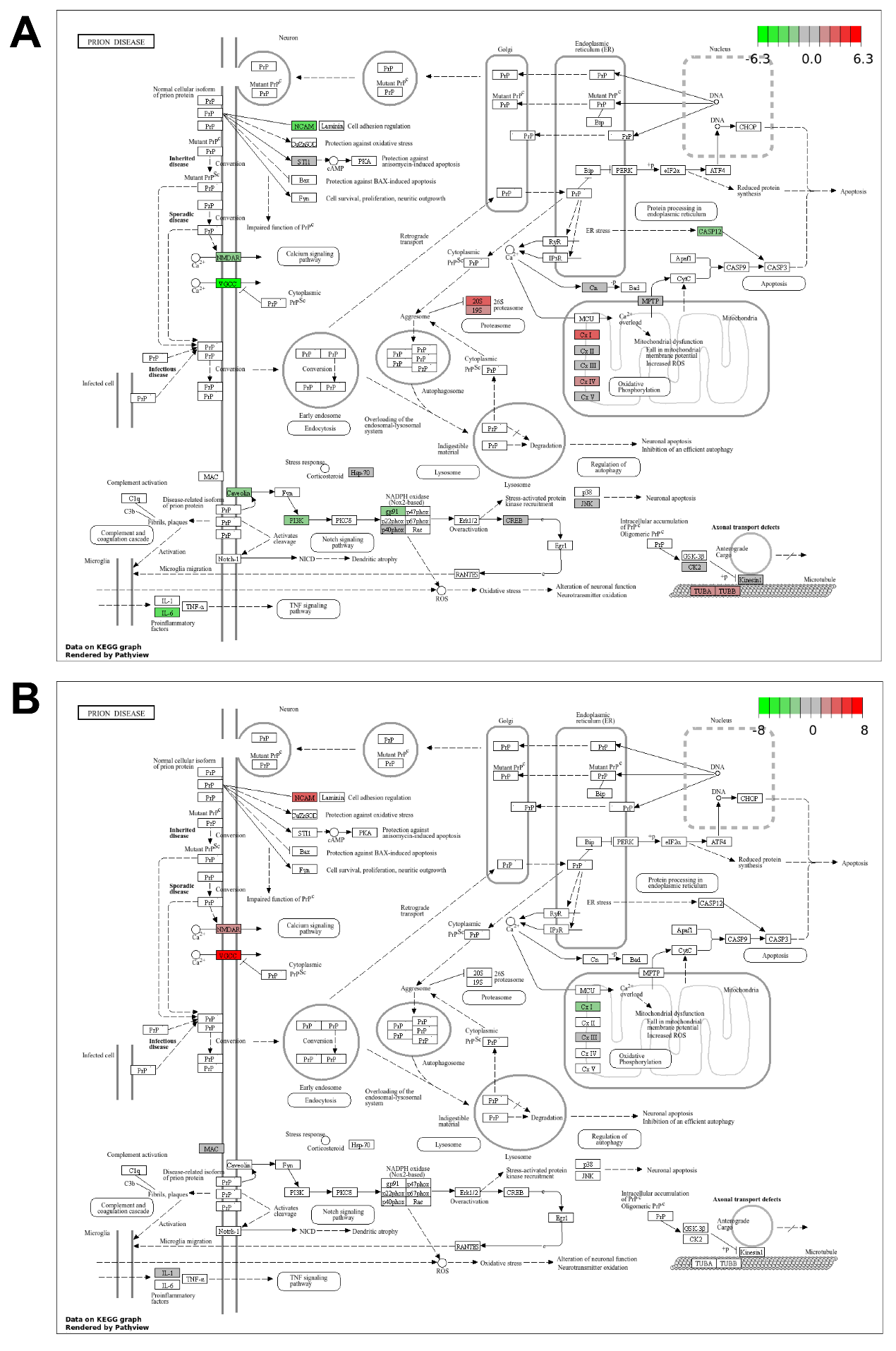


**Figure S3.** The prion disease pathway in the KEGG analysis. (A) HC group vs. NC group. (B) NC group VS. BC group.


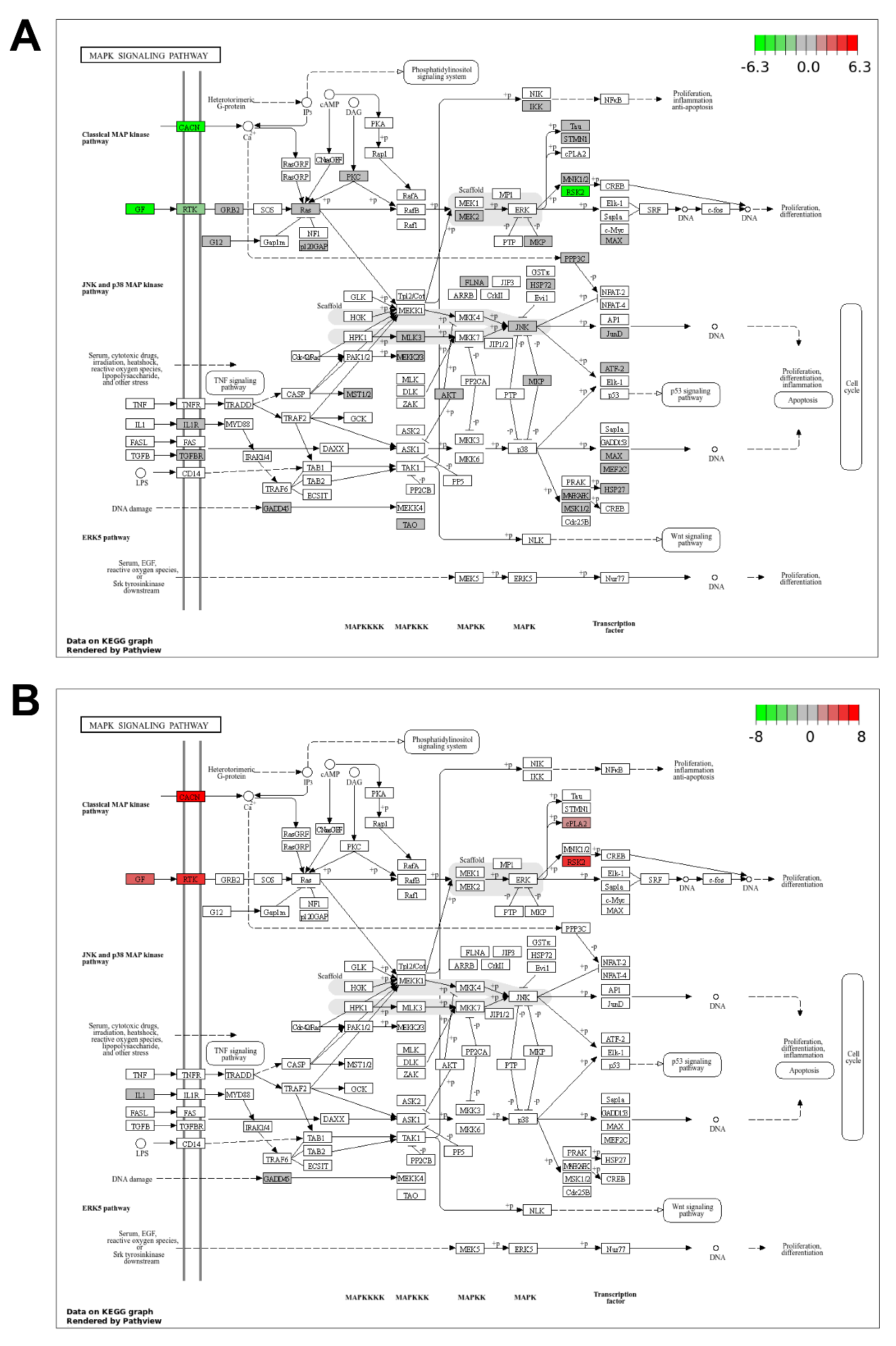


**Figure S4.** The MAPK signaling pathway in the KEGG analysis. (A) HC group vs. NC group. (B) NC group VS. BC group.
